# EEG data and introspective reports from the Libet׳s experiment replication

**DOI:** 10.1016/j.dib.2018.09.066

**Published:** 2018-09-27

**Authors:** Tomáš Dominik, Daniel Dostál, Martin Zielina, Jan Šmahaj, Zuzana Sedláčková, Roman Procházka

**Affiliations:** a Department of Psychology, Palacký University Olomouc, Křížkovského 511/8, Olomouc, Czech Republic; b Department of Medical Ethics and Humanities, 2nd Faculty of Medicine, Charles University in Prague, V Úvalu 84, Prague 5, Czech Republic

## Abstract

This article provides data from a contemporary replication of Libet׳s experiment. For the methodology, results and discussion of the replication, see the article “Libet׳s Experiment: A Complex Replication” (Dominik et al., 2018). Three types of data are presented in this article: (1) introspective reports (M, W and S), (2) EMG onset times relative to a mouse click or to the target time in tasks with a movement at pre-set time and (3) relevant averaged EEG data.

**Specifications table**

**Table:** 

Subject area	*Psychology*
More specific subject area	*Cognitive Psychology – Libet׳s Experiment*
Type of data	*Tables (introspective reports, EMG onsets timing, averaged EEG plots)*
How data was acquired	• *EEG – BIOPAC MP150 unit, EEG100C amplifiers, CAP100C EEG cap* • *EMG – BIOPAC MP150 unit, EMG100C amplifier, EL503 electrodes* • *introspective reports – custom “Libet׳s clock” software*
Data format	• *EEG filtered and epoched* • *readiness potentials onsets analyzed* • *EMG onsets analyzed* • *introspective reports analyzed*
Experimental factors	• *spontaneous movement trials* • *skin stimulation trials* • *performed and vetoed movements at pre-set time trials*
Experimental features	*Eight participants performed four different tasks replicating Libet׳s experiment. First, they performed a spontaneous movement while watching a fast-rotating clock and then indicated the time of the first urge to move (the W report) or the time of perceived movement initiation (the M report). Second, the participants were given a skin stimulus at unknown time and then indicated the time of the stimulus delivery (the S report). Third, the participants were presented a target time on the clock face and performed the movement when the clock reached the target time (P). Fourth, the participants prepared to perform the movement at the target time but then vetoed the movement execution (Pv).*
Data source location	*Olomouc, Czech Republic (N49*°*35*′*46.422*″*, E17*°*15*′*15.667″*)
Data accessibility	*Data is with this article.*
Related research article	[1] T. Dominik, D. Dostál, M. Zielina, J. Šmahaj, Z. Sedláčková, R. Procházka, Libet׳s Experiment: A Complex Replication, Conscious Cogn. 65 (2018) 1–26. https://doi.org/10.1016/j.concog.2018.07.004

**Value of the data**

• Original data from Libet׳s experiment [2], [3], [4] were presented as results only, which were not possible to re-analyze; our replication aims to publish authentic data sample.
• Alternative analysis procedures than those presented in our main article [1] may be applicable; data presented here allow other researchers to approach the analyses from different perspectives.
• Presented data may help other researchers to identify potential problems in methodology and data analysis procedures and avoid them in future Libet-style experiments.

## 1 Data

Data are presented in three Excel files. The first file pertains the introspective reports (Introspective data.xlsx), the second file contains mostly data on the time difference between the EMG activation and the respective mouse click (EMG onsets.xlsx) and the third file presents data needed to construct averaged EEG plots for the respective trials series together with the readiness potential onsets obtained using both the eye-ball inspection (RP_MN_) and RP_90%_ calculation (EEG plots.xlsx).

The Introspective data file is divided into six spreadsheets, each for a respective combination of the series type (M, W or S) and the mode of recall (A or O). Each spreadsheet presents a series of 40 trials (in mode A) or 41 trials (in mode O) in each row. Rows are labeled by following factors: *participant* performing the corresponding series, *session* in which the series was performed, *mode of recall* and *series type*. The *M(A)* and *W(A)* spreadsheets present the M reports (subjective timing of the movement initiation) and the W reports (subjective timing of the urge to move) in each mode A trial, relative either to the mouse click (“resp<>click”) or to the EMG onset (“resp<>EMG”); negative value means that the reported time was before the mouse click or the EMG onset. The *S(A)* spreadsheet shows the S reports (subjective stimulus registration) in each mode A trial relative to the skin stimulus delivery (“resp<>stimulus”); negative value means that the reported time was before the stimulus delivery. The *M(O)*, *W(O)* and *S(O)* spreadsheets contain analogous data, but the reports are averaged for the whole respective series since the O mode of recall does not allow to calculate the reports for individual trials.

The EMG onsets file contains two spreadsheets. The first spreadsheet, called *EMG<>click*, presents separate series in each row, again labeled by *participant, session, mode of recall* and *series type*; in the following columns, it presents the EMG onset time relative to the mouse click (“EMG<>click”) in the M, W and P series; negative value means that the EMG preceded the mouse click. The second spreadsheet, called *Target precision in P series*, presents two types of data for the P series – the “EMG<>target” columns show the time difference between the EMG activation and the target time at which the movement was supposed to be performed (negative value means that the EMG was activated before the target mark); the “click<>target” columns show the time difference between the mouse click and the target time.

The EEG plots file presents two spreadsheets. Common principle is that numbered columns mark milliseconds, while the 0 time marks the EMG onset in the M and W series, skin stimulus delivery in the S series and the target time in the P and Pv series (for instance, column *−1500* in the M series means 1500 ms before the EMG onset); the “valid n” column shows how many valid trials were averaged to constitute the resulting EEG plot. The spreadsheet *Grand Average* presents grand-averaged EEG data for each series type and each electrode (Fp_1_, Fp_2_, C_z_, P_3_, C_3_ and C_4_). The spreadsheet *Individual EEG* presents averaged EEG plots for each *series* and *electrode*. There are also additional columns in the *Individual EEG* spreadsheet pertaining the readiness potential and P300 positivity (for more details on the RP types and methods of RP onset estimation, see Section 2.6 in the main article [1]):

• **RP type** = type of the readiness potential detected using an eye-ball inspection (type I, II, III or pre-set)
• **RP_MN** = readiness potential onset detected using the eye-ball inspection (also called “main negative”, MN)
• **RP_90%_bl <−1500;−1000>** = readiness potential onset calculated using the RP_90%_ method with the baseline set as the mean voltage in the interval <−1500;−1000>
• **RP_90%_bl <-2500;−1500>** = readiness potential onset calculated using the RP_90%_ method with the baseline set as the mean voltage in the interval <−2500;−1500>
• **P300** = if a P300 positivity was detected, 1 is stated in this column

## 2 Experimental design, materials, and methods

The data were obtained from eight participants who performed several experimental tasks. Separate task executions were organized into individual *trials*, each set of 40 or 41 trials constituted a *series*, three consecutively performed series constituted a *session*. Consecutive sessions were performed about a week apart. The series were organized in the sessions so that the experimental conditions were properly rotated (see Section 2.5, Tables 1 and 2 in our main article [1]). Altogether, seven sessions with each participant took place, while the first session was for training purposes only.

There were five types of series: M, W, S, P and Pv.

In the *M series*, the participants were seated in front of a clock face with a dot revolving around the clock face at a fast pace (1 revolution per 2.56 s), while we were recording the EEG (on Fp_1_, Fp_2_, C_z_, P_3_, C_3_ and C_4_; 0.1–35 Hz band pass filter) and EMG (bipolar from musculus extensor indicis; 10–500 Hz band pass filter). The participants were required to click a mouse button whenever they felt like it and then report at what time they realized that the movement begun. This could have been done by two “modes of recall”, which were identical in all series in a single session, but changed in consecutive sessions. In the *absolute* mode of recall (A), the participant simply clicked on a location which was occupied by the dot at the reported time. In the *order* mode of recall (O), the participant stated whether the reported time was before, after or at the time which was being displayed by a static dot on the clock face after the movement was performed. The reports of the movement initiation are referred to as the “M reports”.

In the *W series*, the task is predominantly identical to the M series. The only difference was that in the case of the W series, the reported time was not when the movement begun, but when the participant felt the first urge to move. This report is referred to as the “W report” (from “wanting”).

In the *S series*, the participant was seated in front of the clock face and had a tactile skin stimulator attached to the left wrist. EEG was recorded the same way as in all other series types, but the EMG was not recorded (as it is irrelevant to the S task). The skin stimulator delivered the skin stimuli at unknown time, after which the participant was asked to report the time when he or she registered the stimulus (this is referred to as the S report). Again, this could have been done in either the A or the O mode of recall.

In the *P series* (also called the “pre-set” series in the literature), the clock face presented the moving dot together with a bright green static “target” point. The participant׳s task was to perform the movement when the moving dot reached the target point. No introspective data was then reported; only the EEG data and EMG onsets are of interest to us.

In the *Pv series*, the clock face presented the same arrangement as in the P series. The participant was instructed to prepare to make the movement at the pre-set time, but then “veto” the movement just before it begun. Again, no introspective data was reported; we are interested in the EEG data and EMG onsets only.

For the M, W and S reports, see the Introspective data file. It is important to note that in the A mode of recall, the reports were calculated as the differences between the reported time and the respective mouse click or the EMG onset, while in the O mode of recall, the reports were calculated using a formula presented at the end of Section 2.2 in our main article [1]. The EMG onsets (EMG_0_) were identified in a three-steps procedure:

1. Absolute value of the raw EMG voltage was calculated.
2. The Butterworth procedure was applied to rectify the data (i.e. the integrated EMG was acquired).
3. EMG onset was identified when the integrated EMG level in the segment from 300 ms before the mouse click to 200 ms after the mouse click exceeded a threshold defined as five times the interquartile range of the baseline (spanning from −1500 to −300 ms).

The procedure allowed the possibility that no EMG was found. This is reported in the data files as “no EMG”. See the *EMG onsets* data file for the EMG onsets timing in the individual trials.

The EEG data presented in the *EEG plots* file represent averaged epochs for each series. The averaging procedure followed several steps:

1. The raw EEG data were checked for excessive noise; if it was present, recording for the whole series was rejected (thus it is not present in the *EEG plots* file et al.).
2. EEG data of sufficient quality were additionally band-pass filtered (IIR filter, 0.5–35 Hz).
3. EEG data were temporally locked on the event (as the event we consider the EMG onset in the M and W series, the skin stimulus delivery in the S series and the target point in the P and Pv series) and segmented into epochs spanning from 2500 ms before the event to 800 ms after it.
4. Epochs from the W, M and P series containing no EMG onset or an invalid EMG onset (earlier than −150 ms or later than 0 ms) were rejected, as well as epochs from the Pv series, which contained an EMG onset.
5. Rest of the epochs were averaged into the grand average (see the spreadsheet *Grand Average*) and into the EEG averages for individual series (see the spreadsheet *Individual EEG*).
6. The readiness potential onsets and types were assessed using the two methods described in detail in Section 2.6 in the main article [1].
7. Presence of the P300 positivity was assessed.

## Footnotes

Appendix A Supplementary data associated with this article can be found in the online version at https://doi.org/10.1016/j.dib.2018.09.066.
